# Temperature compensation design and experiment for a giant magnetostrictive actuator

**DOI:** 10.1038/s41598-020-80460-5

**Published:** 2021-01-08

**Authors:** Zhangrong Zhao, Xiaomei Sui

**Affiliations:** 1 Department of Logistics, Beijing Wuzi University, Beijing, 101149 China; 2 Department of Electronic and Information Engineering, North China Institute of Science and Technology, Sanhe, 065201 China

**Keywords:** Mechanical engineering, Techniques and instrumentation

## Abstract

Because the performance of giant magnetostrictive materials (GMMs) can vary at different temperatures, the positioning accuracy of a giant magnetostrictive actuator is affected by heat. In this work, a new simplified control strategy under compulsory water cooling is proposed to maintain a constant GMM temperature. Based on this strategy, a coupled turbulent flow field and temperature field finite element model is created for a GMM smart component. The model is simulated using COMSOL Multiphysics software version 5.3. Through simulations, the temperature field distribution of GMM smart components is analysed under different drive input currents and cooling water flow rates. Based on the obtained simulation results, a GMM intelligent component temperature control device is constructed. The experimental results are in good agreement with the simulation results; a thermostatic control effect is achieved in the thermostat of the giant magnetostrictive rod. Thus, the proposed temperature control strategy is proven effective via simulations and experiments.

## Introduction

Thermal expansion occurs in a giant magnetostrictive material (GMM) when its temperature increases, which can change the magnetostrictive coefficient^1–3^. A GMM has a larger thermal expansion coefficient, and the magnetostriction coefficient is the same order of magnitude; this indicates that the thermal elongation of the GMM will considerably affect the precision of the giant magnetostrictive actuator (GMA) in a negative manner. For GMA applications requiring precise positioning^4–7^, the rise in temperature will have a direct impact on the accuracy of the GMA positioning; therefore, steps must be taken to eliminate or suppress the adverse effects due to the increasing temperature.

Currently, the two primary methods used to suppress GMA thermal deformation are passive compensation and active temperature control. In passive compensation, the axial position change of a flexible support mechanism compensates for the thermal deformation of the GMM rod. Liu^8^ proposed a thermal deformation passive compensation mechanism. The compensation sleeve generated thermal expansion and moved downward under the action of heat, which drove the lower magnetic block and the GMM rod to move downward. Through the experiment, under the condition of a lower frequency input current, the thermal deformation compensation mechanism completed the thermal deformation compensation caused by the GMA to the rise in temperature. However, under a higher frequency input current, GMA temperature compensation can induce errors. Xia^9^ designed a fine-tunable thermal deformation compensation mechanism consisting of a flexible hinge lever support structure. With a 10 Hz input current, the maximum thermal deformation output of the GMA was less than 0.5 µm, and with a 150 Hz input current, the maximum thermal deformation output was less than 3.5 µm. This compensation mechanism had a complicated structure, a large radial volume and a large number of parts. A real-time compensation mechanism for thermal deformation was designed by Yu^10^. The basic principle was to use the leaking oil of a nozzle valve to flow through the GMM rod and the thermal compensation tube. The thermal deformation of the thermal compensation tube would offset the thermal elongation of the GMM rod due to the increase in oil temperature to reduce the impact of thermal deformation on the controllable displacement output. The actual thermal compensation effect of the compensation mechanism of the GMA nozzle flapper valve was tested during winter and summer under different input currents. At the beginning of the experiment, the temperature rose quickly, but when the temperature reached a certain value, the temperature rose slowly.

Thermal expansion compensation can effectively offset the thermal deformation of a GMM rod; however, an appropriate inner material must be selected during the design process. Yamamoto^11^ introduced a double-sleeve thermal compensation mechanism. The inner sleeve was made of stainless steel with very low magnetic permeability. Because its thermal expansion coefficient was equivalent to the GMM, the thermal deformation of the GMM rod was compensated by the thermal elongation of the inner sleeve. Wang^12^ designed a thermal compensation structure to offset the thermal expansion deformation of a GMM. Because the upper end of the compensation sleeve was connected to the upper cover and belonged to the fixed end, the lower end was connected with a lower pure iron sheet, and there was a gap between the bases and the lower end that moved down, so the compensation sleeve would move down due to thermal expansion caused by heat. A test showed that the experimental compensation value was 2.3 µm at 25 °C, the theoretical thermal expansion value was 2.5 µm, and the compensation error rate was 8%. The experimental compensation value was 17.6 µm at 60 °C, the theoretical thermal expansion value was 20 µm, and the compensation error was 12%. The low temperature (below 45 °C) compensation effect was better than the high temperature effect.

Passive compensation mechanisms are complex and cannot fundamentally eliminate thermal errors. The active temperature control method uses a cooling measure to assure the GMA works at a constant temperature. The traditional forced water temperature control method uses water as the cooling medium. The water cooling system for GMMs has been applied to GMAs^13^. A water cooling pipe was designed between the coil and GMM rod. When the current is less than 1 A, cooling water can remove heat from the coil and GMM rod, and the water cooling system works effectively. However, when the current is greater than 1.5 A, the rise in temperature of the GMM rod is considerable, and the water cooling system cannot ensure a constant GMM rod temperature. Jia^14^ used forced water cooling to eliminate the influence of coil heating on a giant magnetostrictive microdisplacement actuator. Through the feedback of the temperature sensor, the change in the water temperature was input into a computer, and a computer temperature control circuit was used to control the heating device so that the temperature of the water flowing into the cooling pipe was controlled at 30 ± 0.01 °C. By forced water cooling, the temperature in the driver was kept constant, thereby eliminating the influence of heat on the driving coil current. Experiments showed that the giant magnetostrictive microdisplacement actuator could reach a displacement resolution of 0.5 nm, and the displacement range could reach 40 µm. Lu^15,16^ designed a special water temperature maintenance system for a GMA, controlling the water temperature through a computer and passing a certain temperature of cooling water through the GMA to ensure that the GMM worked at a constant temperature. Through experiments, when the GMA input current was 0.8 A and below, the output displacement remained stable over time, and its temperature control effect was remarkable. When there is a larger current input, the temperature slowly increased over time. When the input current was 1.5 A, the displacement increased by 0.1 µm within 60 min. When the input current was 2.0 A, the displacement increased by 0.51 µm within 60 min, and the trend showed a continued increase. This shows that the design of forced water cooling needs to control not only the water temperature but also the water velocity and flow rate.

Water flows through the drive coil to reduce the coil heat, which prevents the flow of heat from the coil to the GMM and ensures a constant GMM working temperature, thus eliminating thermal deformation. The semiconductor refrigeration temperature control method places the cold side of a semiconductor cooler in the drive coil bobbin, and then the coil heat is absorbed by the semiconductor cooler^17–19^. The semiconductor cooler cycles the cooling water to cool the hot side and enhance the cooling effect of the hot end. Xu^18^ installed several semiconductor refrigerators at both ends of a GMA coil bobbin and coated the contact surface with thermally conductive silicone grease to increase the thermal conductivity. Cooling water was passed through the hot end of the refrigerator to enhance the heat dissipation effect and ensure higher cooling efficiency while keeping the temperature of the hot end stable. With increasing work time, the temperature of the radial centre point of the coil bobbin first increased from 20 °C to approximately 30 °C, then dropped to approximately 19 °C, and finally fluctuated at 20 ± 1 °C.

The phase change temperature control method has been recently introduced into GMA temperature control applications^20–23^. Liu^20^ placed a phase change material between the drive coil and the magnetostrictive rod, which can absorb the heat generated by the coil and prevent this heat from being transferred to the magnetostrictive rod while absorbing the magnetostrictive rod itself due to eddy current heat generated by hysteresis loss. The test results showed that the phase change temperature control device could control the temperature of the giant magnetostrictive rod at 45 ± 0.5 °C. The displacement output error caused by the working temperature change did not exceed 0.1 µm, and the corresponding output of the giant magnetostrictive actuator accuracy was 0.5 µm.

Using semiconductor refrigeration to cool a GMA requires specialized semiconductor refrigeration, which makes the GMA structure more complex. In addition, phase change temperature control requires a complex process of phase change material selection. Due to the limited heat absorption of phase change materials, for high-power GMAs under long-term working conditions, the use of phase change materials to control the temperature of a GMM rod is not effective. Zhang^24^ proposed a GMM temperature-stretching capacity characteristic model for a GMA and realized the temperature compensation of its expansion capacity. The experimental results showed that the temperature characteristic compensation could reduce the output error of the GMM from 1.5 to 0.4 µm. To address a GMA being heated under high-frequency and high-current driving seriously affecting its effective displacement output accuracy, Ji^25^ used a tubular cooling structure measures were used to suppress the increase in actuator temperature. Tube cooling provided a better cooling effect, and the temperature of the magnetostrictive rod could be controlled within 50 °C. Kwak^26^ studied a magnetostrictive actuator with built-in compressed air to dissipate heat and circulated cold air compressed at an appropriate temperature and speed to keep the temperature of the GMM rod constant. Experiments showed that the temperature of the GMM rod was kept at approximately 20 °C, and the displacement of the actuator was kept at 5 µm. Zhao^27^ used cooling oil with insulating properties to directly flow through the GMA drive coil to cool the heat generated by the coil. Compared with the use of cooling water that required special pipes, the use of cooling oil could appropriately reduce the GMA volume. Bo^28^ proposed a new type of water immersion cooling system for a GMA, which increased the space utilization by approximately 5% and increased the effective contact space by approximately 4.32 ml. The research results showed that the coolant flow rate in the cooling system had an obvious inhibitory effect on the actuator, and the pressure at the inlet and outlet of the coolant and the temperature of the actuator basically had no inhibitory effect.

In summary, for low-power GMA systems, due to space and cost reasons, passive compensation methods are typically used to offset thermal deformation by using components with approximately the same thermal expansion coefficient as a GMM. The design components are more complicated, and the compensation effect is good for low temperatures, but the compensation error is larger for high temperatures. For high-power or high-precision GMA systems, an active temperature control system is typically used to ensure a constant temperature when a GMM rod is working to eliminate thermal errors. Traditional water-cooling systems have shown through experiments that they can maintain GMM temperature better at low frequencies or low temperatures, but at high frequencies or high temperatures, the GMM temperature fluctuates greatly. The traditional forced water-cooled temperature control method is complex; not only does the tank temperature need to be controlled, but the pump flow method must also be adjusted according to the GMA temperature changes.

In this work, a new simplified forced water cooling control strategy is proposed to maintain a constant GMM temperature. Based on this goal, a coupled turbulent flow field and temperature field finite element model is created for the GMM smart component. The model is simulated using COMSOL Multiphysics software version 5.3. The temperature control strategy is verified as effective via simulations and experiments.

## Structure of the smart component

**Figure 1:**
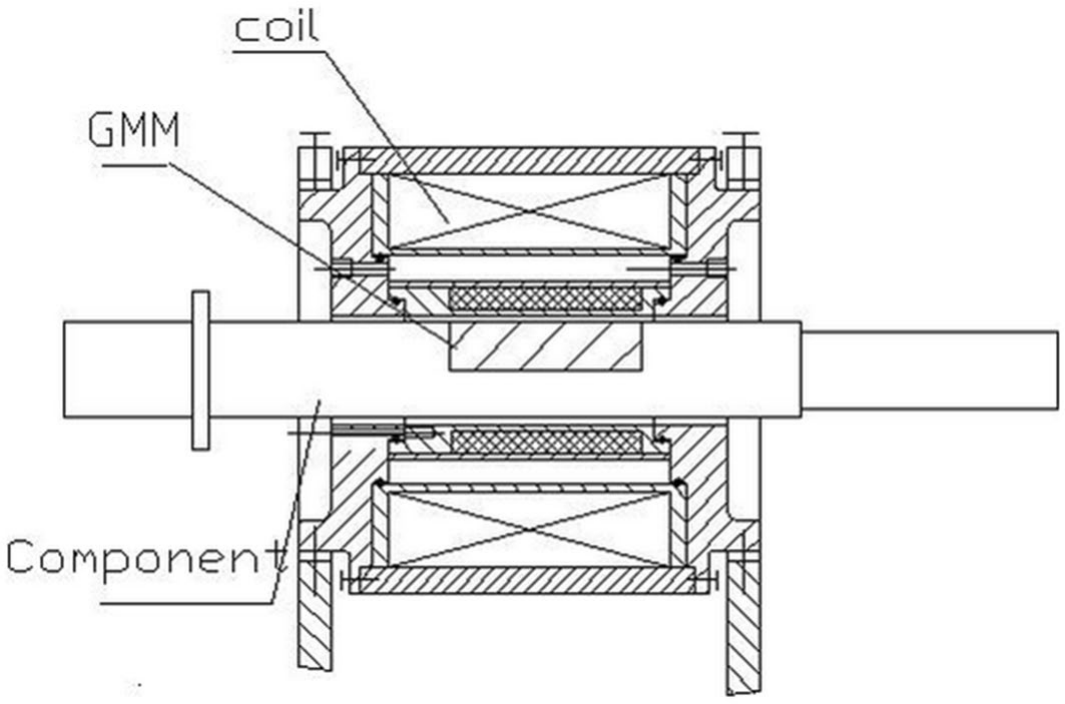
Structure of the smart component.

A structure for machining a non-cylindrical pin hole has been proposed^29^ wherein the GMM is embedded into the component. Under an applied current, the GMM elongates to bend the smart component and generates a radial feed. Figure 1 shows the structure of the smart component.

**Figure 2:**
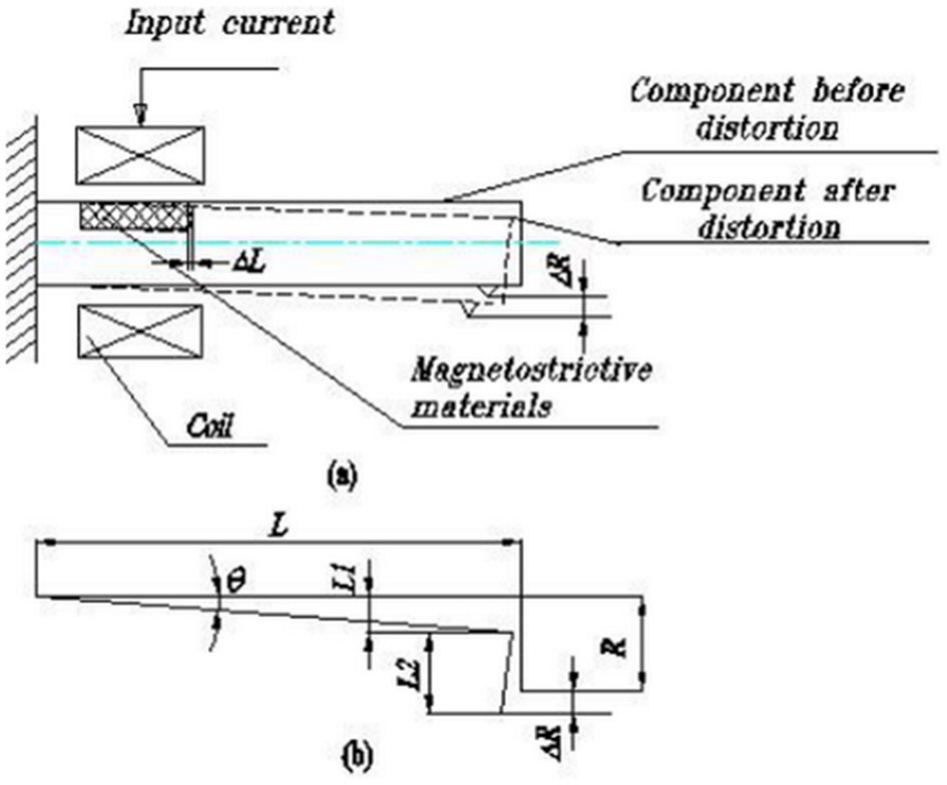
Principle of smart component embedded GMM: (**a**) embedded GMM smart components (**b**) zoomed view of the component corner.

Figure 2 shows the working principle of the smart component. The GMM magnetostrictive $\lambda$ and driving magnetic field satisfy a certain function $\lambda = F(H)$. The current of the driving coil and magnetic field approximately satisfy $H = nI$. Therefore, we can discern that the magnetostrictive current of the driving coil satisfies the function $\lambda = F(nI)$. The component bending results in a rotating turn angle $\theta$. In Fig. 2b, we can obtain the following equations:

$$ \begin{aligned} L_{1} & = L\sin \theta \\ L_{2} & = R\cos \theta \\ \Delta R & = L_{1} + L_{2} - R = L\sin \theta + R\cos \theta - R \\ \end{aligned} $$

Because the rotating angle $\theta$ is considerably small, we can assume that $\sin \theta = \theta$, $\cos \theta = 1$; therefore, $\Delta R = L\theta$. Rotating angle $\theta$ is proportional to $\Delta L$, i.e. $\theta = K\Delta L$, where *K* is the scale coefficient. Thus, the radial increment $\Delta R = KL\Delta L$ is obtained. From the magnetostrictive $\lambda = \Delta L/L^{\prime }$, where $L^{\prime }$ is the length of GMM, we can see that radial increment and current satisfy the equation $\Delta R = KLL^{\prime } F(nI)$. According to the relationship between the radial increment and current, we can see that only the current of the driving coil needs to be controlled. Thus, we can obtain the required radial displacement and implement the machining non-cylindrical pin hole. Table 1 shows the design parameters of GMM smart components.

**Table 1:** Design parameters of GMM smart component.

Diameter of component (mm)	Length of component (mm)	Diameter of GMM rod (mm)	Length of GMM rod (mm)	Inner diameter of driving coil (mm)	Outter diameter of driving coil (mm)
30	310	30	60	78	146

**Figure 3:**
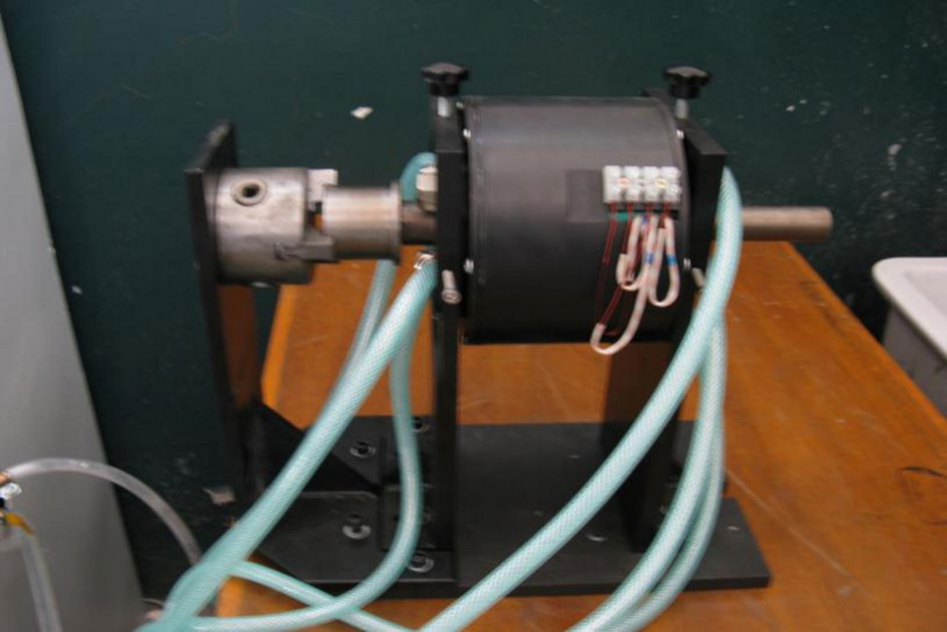
Smart component system.

Figure 3 shows the structural design of the smart component, where the number of windings is 1,365.

**Figure 4:**
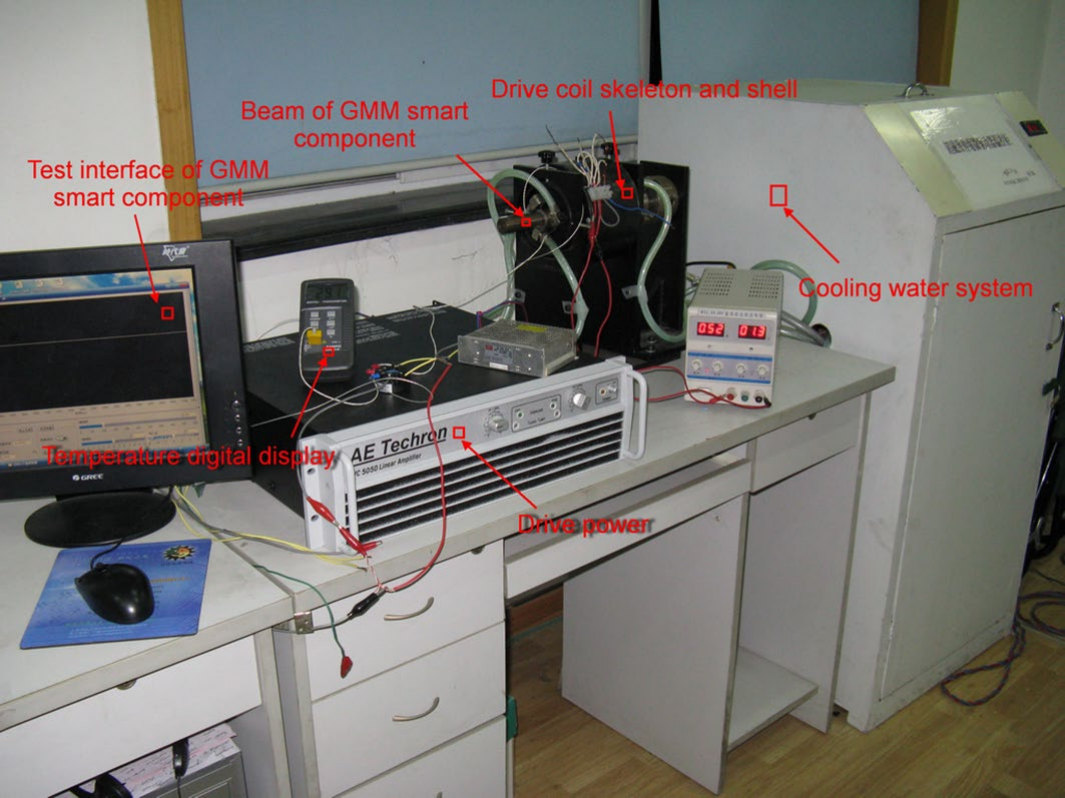
Smart component testing system.

Figure 4 shows the smart component testing system, which includes the forced water cooling control system, smart component system and input signal control system.Before syetem begins to work, cooling water is heated to a certain temperature,then the cooling water is injected into smart component at a certain flow rate.The smart component output displacement, drive magnetic field, GMM rod temperature and input current are attainted by various detctors respectively.

## Model of the GMM smart component

This is a typical fluid model for convective heat transfer. Thus, it is necessary to establish a model involving fluid coupled heat transfer calculations.

### Time-averaged movement equations of turbulence and convection

It can be assumed that flowing water is in a turbulent state based on the structural parameters of the GMM smart component. Therefore, the temperature distribution of the GMM smart component is calculated by using the Reynolds-averaged Navier–Stokes equation (RANS). In the case of turbulent motion, the instantaneous variables of a fixed point ($\phi$) can be divided into the average ($\overline{\phi }$) and pulsation ($\phi^{\prime }$) with $\phi = \overline{\phi } + \phi^{\prime }$; furthermore, the time average of $\phi^{\prime }$ is zero. The average ($\overline{\phi }$) follows these calculation rules ^28^:

1 $$ \left\{ {\begin{array}{*{20}l} {\overline{\overline{f}} = \overline{f}} \hfill & {\overline{f}^{\prime} = 0} \hfill \\ {\overline{f + g} = \overline{f} + \overline{g}} \hfill & {\overline{{\overline{f} \cdot \overline{g}}} = \overline{f} \cdot \overline{g}} \hfill \\ {\overline{{\overline{f} \cdot g}} = \overline{f} \cdot \overline{g}} \hfill & {\overline{f \cdot g} = \overline{f} \cdot \overline{g} + \overline{{f^{\prime } g^{\prime } }} } \hfill \\ {\frac{{\overline{\partial f} }}{\partial s} = \frac{{\partial \overline{f}}}{\partial s}} \hfill & {\overline{\alpha f} = \alpha \overline{f}} \hfill \\ \end{array} } \right. $$

where $f$ and $g$ are two physical quantities, $s$ represents any independent variable, and $\alpha$ is a constant. The time-averaged movement equations of incompressible turbulence can be obtained from Eq. (1).

### Continuity equation

Equation (2) is the continuity equation for incompressible flow.Three variables below(u, v, w) are the velocity component of the particle in the fluid in the X, Y, and Z directions respectively.

2 $$ \frac{\partial u}{{\partial x}} + \frac{\partial v}{{\partial y}} + \frac{\partial w}{{\partial z}} = 0 $$

where $u = \overline{u} + u^{\prime }$, $v = \overline{v} + v^{\prime }$, $w = \overline{w} + w^{\prime }$, and Eq. (2) can be substituted by Eq. (3):

3 $$ \frac{{\partial \overline{u}}}{\partial x} + \frac{{\partial \overline{v}}}{\partial y} + \frac{{\partial \overline{w}}}{\partial z} + \frac{{\partial u^{\prime}}}{\partial x} + \frac{{\partial v^{\prime}}}{\partial y} + \frac{{\partial w^{\prime}}}{\partial z} = 0 $$

Using Eq. (1), the time-averaged equation of Eq. (3) can be described as:

4 $$ \frac{{\partial \overline{u}}}{\partial x} + \frac{{\partial \overline{v}}}{\partial y} + \frac{{\partial \overline{w}}}{\partial z} = 0 $$

5 $$ \frac{{\partial u^{\prime } }}{\partial x} + \frac{{\partial v^{\prime } }}{\partial y} + \frac{{\partial w^{\prime } }}{\partial z} = 0 $$

Equations (4) and (5) are the continuity equations for incompressible turbulence. We can find the time-averaged turbulent velocity satisfying the continuity equation.

### Movement equation

The average and pulsation are substituted into the Navier–Stokes equation, and then the time-average is calculated. Thus, the following equation can be obtained:

6 $$ \frac{{\partial (\rho \overline{u}_{i} )}}{\partial t} + \frac{{\partial (\rho \overline{u}_{i} \overline{u}_{j} )}}{{\partial x_{j} }} = - \frac{{\partial \overline{p}}}{{\partial x_{i} }} + \frac{\partial }{{\partial x_{j} }}\left( {\mu \frac{{\partial \overline{u}_{i} }}{{\partial x_{j} }} - \rho \overline{{u^{\prime}_{i} u^{\prime}_{j} }} } \right),\quad (i,j = 1,3) $$

where $\rho$ is the fluid density. Equation (6) is the time-averaged movement equations of incompressible turbulence.

### Energy equation

Omitting the dissipated energy items from the incompressible fluid energy equation and assuming every physical quantity is represented by the sum of the average and pulsation, we can deduce Eq. (7):

7 $$ \rho c\frac{{\overline{{\partial (\overline{T} + T^{\prime } )}} }}{\partial t} + \rho c\overline{{(\overline{u} + u^{\prime } )\frac{{\partial (\overline{T} + T^{\prime } )}}{\partial x}}} + \rho c\overline{{(\overline{v} + v^{\prime } )\frac{{\partial (\overline{T} + T^{\prime } )}}{\partial y}}} + \rho c\overline{{(\overline{w} + w^{\prime } )\frac{{\partial (\overline{T} + T^{\prime } )}}{\partial z}}} = \lambda \left[ {\overline{{\frac{{\partial^{2} (\overline{T} + T^{\prime } )}}{{\partial x^{2} }} + \frac{{\partial (\overline{T} + T^{\prime } )}}{{\partial y^{2} }} + \frac{{\partial^{2} (\overline{T} + T^{\prime } )}}{{\partial z^{2} }}}} } \right] $$

By using Eq. (1), Eq. (7) can be transformed as:

8 $$ \rho c\frac{{\partial \overline{T}}}{\partial t} + pc\overline{u}\frac{{\partial \overline{T}}}{\partial x} + \rho c\overline{v}\frac{{\partial \overline{T}}}{\partial y} + \rho c\overline{w}\frac{{\partial \overline{T}}}{\partial z} = \lambda \left( {\frac{{\partial^{2} \overline{T}}}{{\partial x^{2} }} + \frac{{\partial^{2} \overline{T}}}{{\partial y^{2} }} + \frac{{\partial^{2} \overline{T}}}{{\partial z^{2} }}} \right) - \frac{\partial }{\partial x}\left( {\rho c\overline{{u^{\prime } T^{\prime } }} } \right)\; - \frac{\partial }{\partial y}\left( {\rho c\overline{{v^{\prime } T^{\prime } }} } \right) - \frac{\partial }{\partial z}\left( {\rho c\overline{{w^{\prime } T^{\prime } }} } \right) $$

Equation (8) is the energy equation of incompressible turbulence.

### *K* − *ε* turbulent model

The continuity equation, movement equation, and energy equation are not closed equations; therefore, we must consider the relationship between additional pulsation and time-average items. According to the instantaneous Navier–Stokes relationship given by Eq. (9) and Reynolds time-average given by Eqs. (10, 11) ($K$ Equation) can be obtained ^30^.

9 $$ \rho \left( {\frac{{\partial u_{i} }}{\partial t} + u_{k} \frac{{\partial u_{i} }}{{\partial x_{k} }}} \right) = - \frac{{\partial \overline{p}}}{{\partial x_{i} }} + \frac{\partial }{{\partial x_{k} }}\left[ {\mu \left( {\frac{{\partial u_{i} }}{{\partial x_{k} }} + \frac{{\partial u_{k} }}{{\partial x_{i} }}} \right)} \right] $$

10 $$ \rho \left( {\frac{{\partial \overline{u}_{i} }}{\partial t} + \overline{u}_{k} \frac{{\partial \overline{u}_{i} }}{{\partial x_{k} }}} \right) = - \frac{{\partial \overline{p}}}{{\partial x_{i} }} + \frac{\partial }{{\partial x_{k} }}\left[ {\mu \left( {\frac{{\partial \overline{u}_{i} }}{{\partial x_{k} }} + \frac{{\partial \overline{u}_{k} }}{{\partial x_{i} }}} \right) - \rho \overline{{u_{i}^{\prime } u_{k}^{\prime } }} } \right] $$

11 $$ \rho \frac{\partial K}{{\partial t}} + \rho \overline{u}_{j} \frac{\partial K}{{\partial x_{j} }} = \frac{\partial }{{\partial x_{j} }}\left\{ {\left( {\mu + \frac{{\mu_{t} }}{{\sigma_{k} }}} \right)\frac{\partial K}{{\partial x_{j} }}} \right\} + \mu_{t} \frac{{\partial \overline{u}_{i} }}{{\partial x_{j} }}\left( {\frac{{\partial \overline{u}_{i} }}{{\partial x_{j} }} + \frac{{\partial \overline{u}_{j} }}{{\partial x_{i} }}} \right) - c_{D} \rho \frac{{K^{{{\raise0.7ex\hbox{$3$} \!\mathord{\left/ {\vphantom {3 2}}\right.\kern-\nulldelimiterspace} \!\lower0.7ex\hbox{$2$}}}} }}{l} $$

where $\sigma_{k}$ is the pulsation momentum's Prandtl parameter. After using Eq. (11) to determine the fluid viscosity coefficient ($\mu_{t}$), the continuity equation, movement equation, energy equation, and $\varepsilon$ Eq. (12) are closed equations^31^.

12 $$ \rho \frac{\partial \varepsilon }{{\partial t}} + \rho \overline{u}_{j} \frac{\partial \varepsilon }{{\partial x_{j} }} = \frac{\partial }{{\partial x_{j} }}\left[ {\left( {\mu + \frac{{\mu_{t} }}{{\sigma_{\varepsilon } }}} \right)\frac{\partial \varepsilon }{{\partial x_{j} }}} \right] + \frac{{c_{1} \varepsilon }}{K}\mu_{t} \frac{{\partial \overline{u}_{i} }}{{\partial x_{k} }}\left( {\frac{{\partial \overline{u}_{i} }}{{\partial x_{k} }} + \frac{{\partial \overline{u}_{k} }}{{\partial x_{i} }}} \right) - \frac{{c_{2} \rho \varepsilon^{2} }}{K} $$

Using the $K - \varepsilon$ model, the fluid viscosity coefficient is shown as Eq. (13) with the parameters listed in Table 2.

13 $$ \mu_{t} = c_{\mu }^{\prime } \rho K^{\frac{1}{2}} l = (c_{\mu }^{\prime } c_{D} )\rho K^{2} \frac{1}{{c_{D} K^{{{\raise0.7ex\hbox{$3$} \!\mathord{\left/ {\vphantom {3 2}}\right.\kern-\nulldelimiterspace} \!\lower0.7ex\hbox{$2$}}}} }} = c_{\mu } \rho K^{2} {/}\varepsilon $$

**Table 2:** $K - \varepsilon$ model parameters.

*c*_*µ*_	$c_{1}$	$c_{2}$	$\sigma_{k}$	$\sigma_{\varepsilon }$	$\sigma_{T}$
0.09	1.44	1.92	1.0	1.3	0.9–1.0

Thus, the turbulent convective heat transfer can be calculated by solving the continuity equation, movement equation, energy equation, $K - \varepsilon$ turbulent equations, and Eq. (13).

## Results and discussion

### Theoretical model simulation

**Figure 5:**
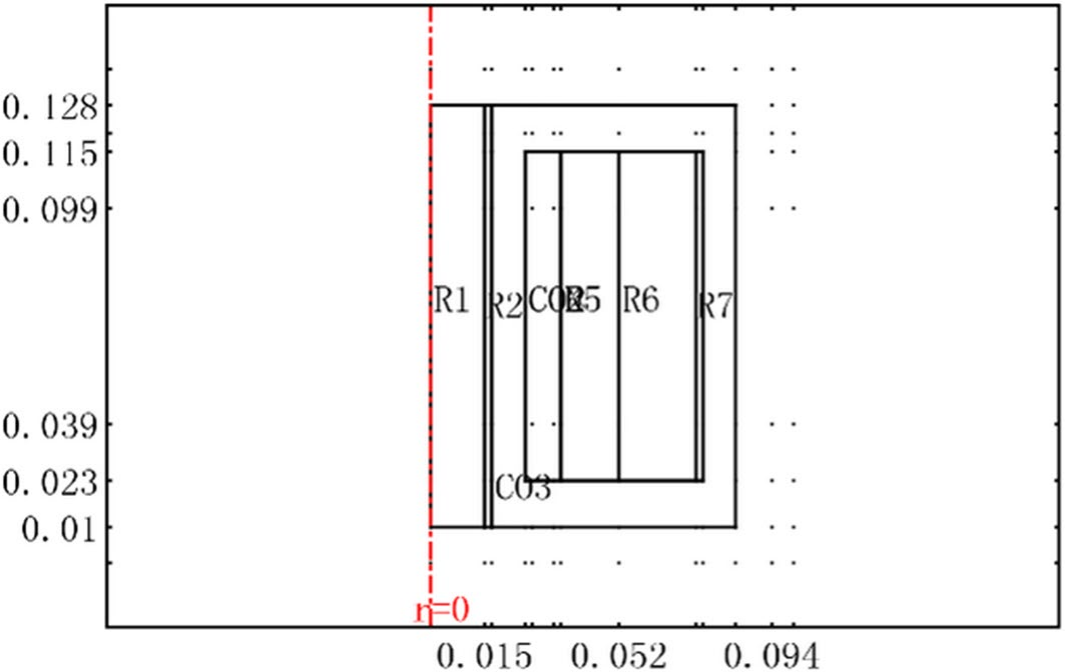
Thermal analysis model of the GMM smart component.

The difference between the GMM and bulk material is ignored; thus, the thermal structure of the GMM can be considered axially symmetric, as shown in Fig. 5.

In the thermal field analysis of the GMM smart component, the key goal is to conduct an analysis of the cooling water’s flow-thermal coupling. Through the theoretical analysis discussed in the previous section, Eqs. (4)–(13) must be solved.

COMSOL Multiphysics software version 5.3 is a professional finite element numerical analysis package. Using this software, modelling the coupling analysis of physics field combinations can be implemented simultaneously by selecting different modules. For a temperature field analysis of the GMM smart component, we used one “$K - \varepsilon$ turbulent model” module and two “General Heat Transfer” modules.

At the inlet, the model is set the velocity component in the z direction, u, to 0.0464 m/s, and that in the r direction, v, to zero. The model is specified a constant pressure value of 0 at the outlet and logarithmic wall functions at the solid walls.The model uses two heat transfer application modes: one for the solid and one for the fluid.These are connected through a heat flux boundary condition, the thermal wall function.

**Figure 6:**
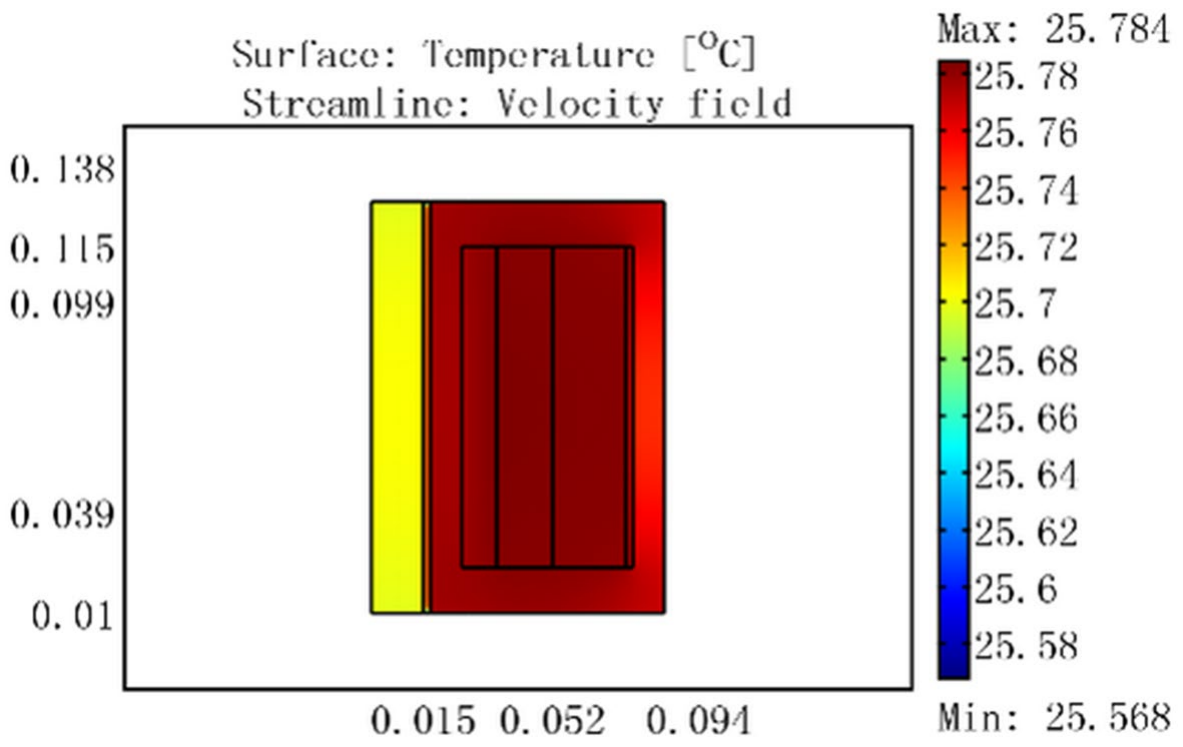
For a current of 0.5 A, temperature distribution of GMM smart component under no cooling water.

**Figure 7:**
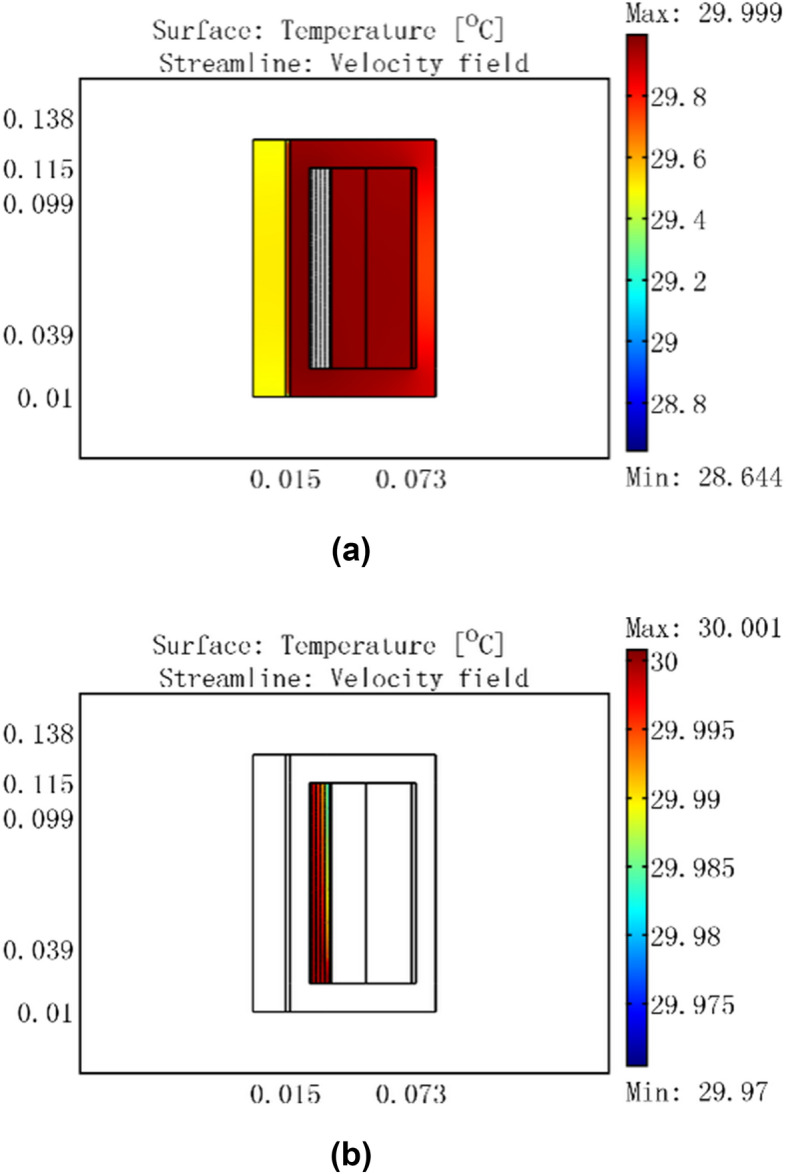
Temperature distribution of GMM smart component for a current of 0.5 A, cooling water temperature of 30 °C, and flow rate of 0.0464 m/s.

**Figure 8:**
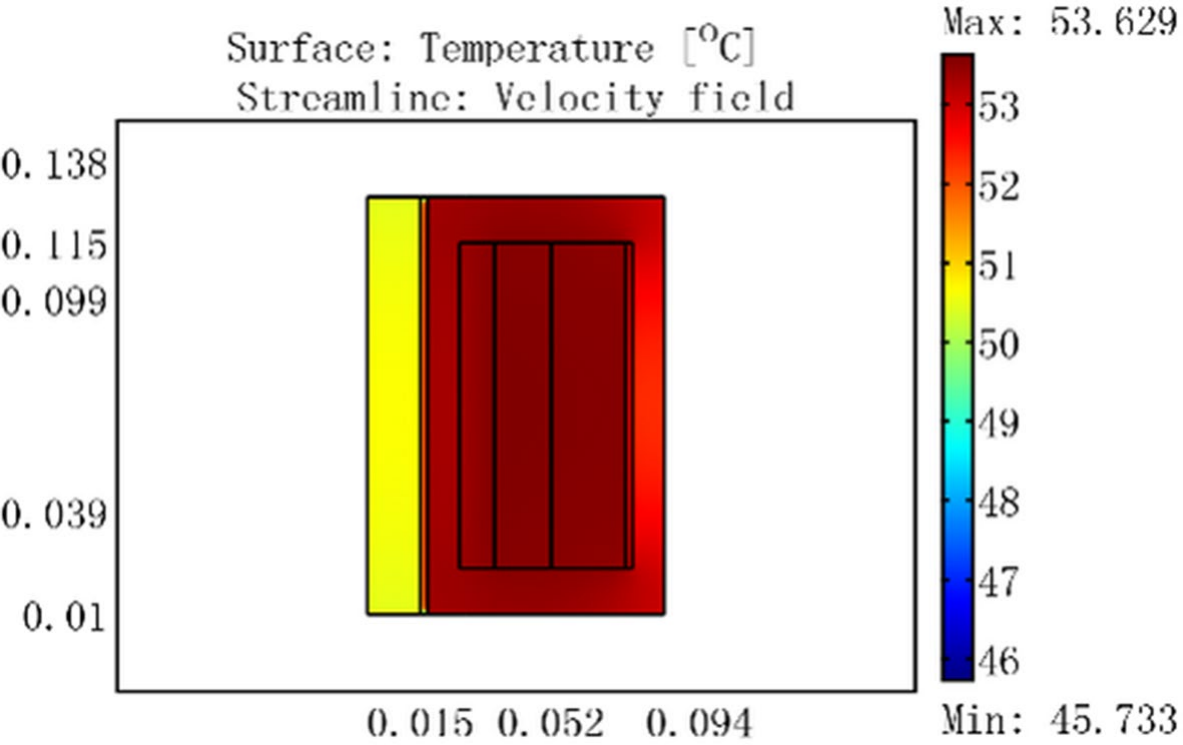
For a current of 3 A, temperature distribution of GMM smart component with no cooling water.

**Figure 9:**
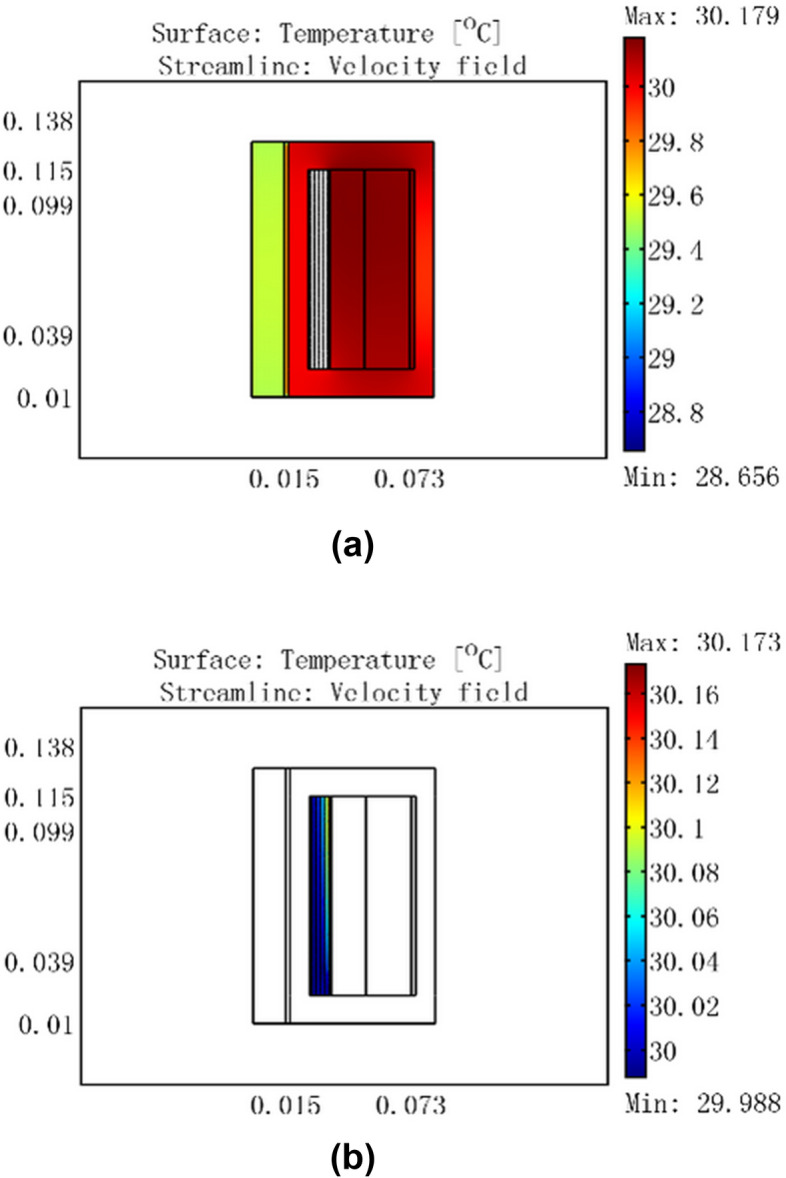
Temperature distribution of GMM smart component for a current of 3 A, cooling water temperature of 30 °C, and flow rate of 0.0464 m/s.

Based on the flow-thermal coupling finite element models, temperature field simulation analysis was completed at different inputs under an ambient temperature of 25 °C. The simulation results are shown in Figs. 6, 7, 8, 9.

Figure 6 shows that the coil temperature rose to a maximum of 25.784 °C and the GMM rod temperature rose to 25.7 °C for a 0.5 A current with no cooling water. When 30 °C cooling water passed through the GMM smart component, because the water temperature was higher than that of the coil, the coil temperature rose to 30 °C and the GMM rod temperature rose to 29.5 °C in Fig. 7a. Figure 7b shows that the inlet water temperature was 30 °C, whereas the temperature at the outlet near the coil was slightly lower at 29.985 °C, which was nevertheless significantly higher than 25.784 °C. This indicates that the heat was transferred from 30 °C cooling water to the coil.

Figure 8 shows that the coil temperature rose to a maximum of 53.629 °C and the GMM rod temperature rose to 50.5 °C for a 3 A current with no cooling water. When 30 °C cooling water passed through the GMM smart component, the coil maximum temperature was 30 °C, the GMM rod temperature was 29.5 °C, and the container wall temperature was 30 °C, as shown in Fig. 9a. Figure 9b shows that the inlet water temperature was 30 °C, whereas the temperature at the outlet near the coil was 30.08 °C. This indicates that heat was transferred from the coil to the 30 °C cooling water, which ensured that the GMM rod temperature remained constant at 29.5 °C.

From the above analysis, it is clear that the GMM rod temperature stayed at 29.5 °C as long as the cooling water temperature was 30 °C for an input current of 3 A or less and a flow rate of 0.0464 m/s. This temperature control can ensure that the GMM remains at a certain temperature, which can eliminate the effects of the drive coil heating for the precise positioning of the GMM smart component.

**Figure 10:**
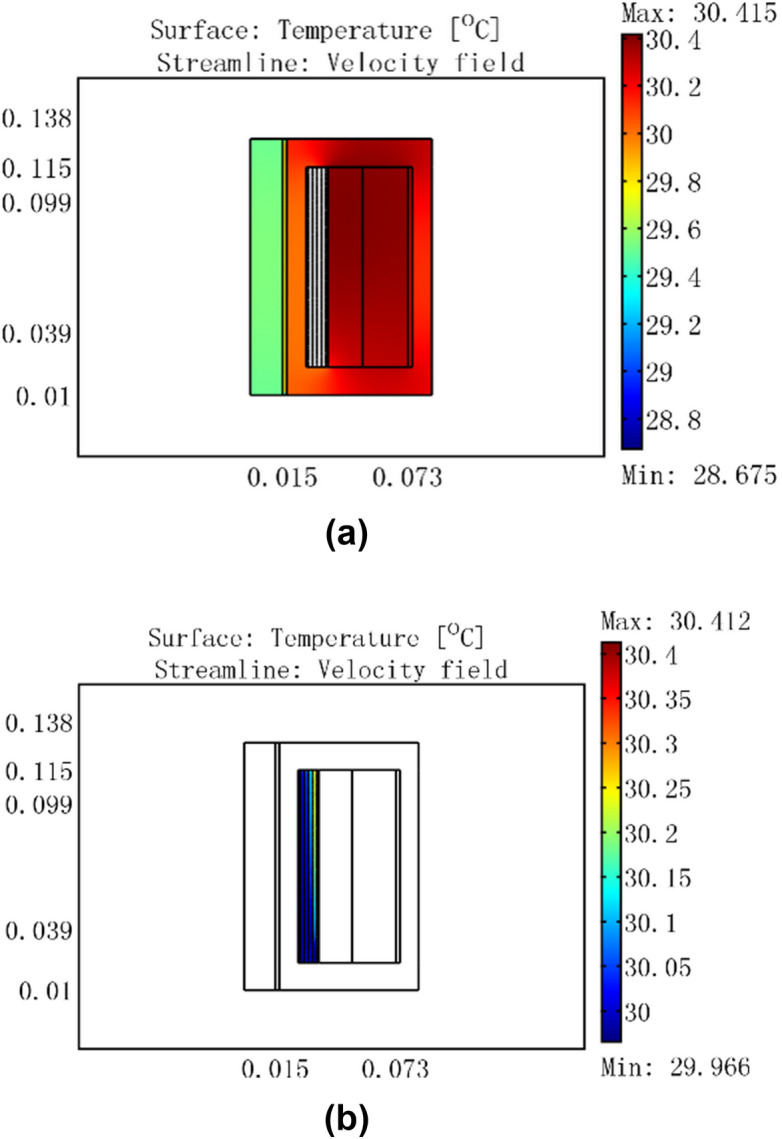
GMM smart component temperature distribution for a current of 3 A, cooling water temperature of 30 °C, and flow rate of 0.01 m/s.

**Figure 11:**
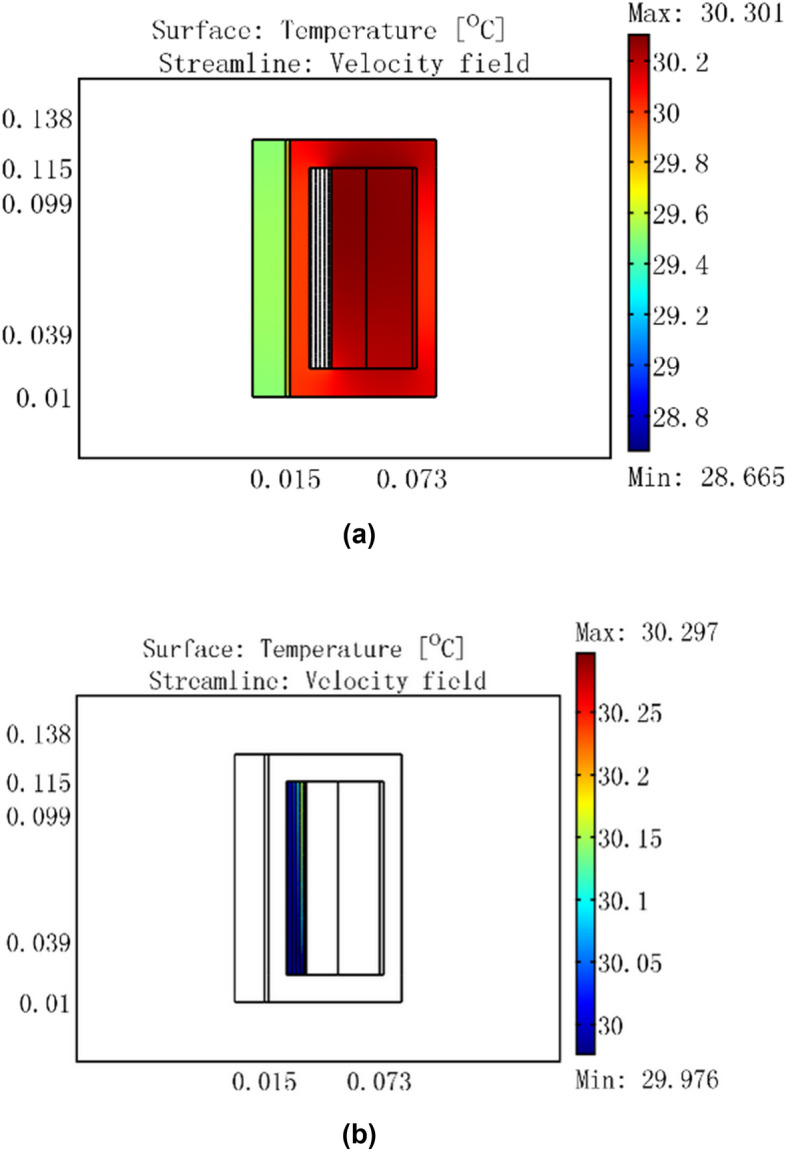
GMM smart component temperature distribution for a current of 3 A, cooling water temperature of 30 °C, and flow rate of 0.02 m/s.

For a cooling water temperature of 30 °C, input current of 3 A, and cooling water input flow rates of 0.01 m/s and 0.02 m/s, the temperature distribution of the GMM smart component is shown in Figs. 10 and 11, respectively.

Figures 9, 10, 11 show that when the input flow rate of the cooling water was 0.0464 m/s, the GMM rod temperature was 29.5 °C; when the input flow rate of the cooling water was 0.01 m/s, the GMM rod temperature was 29.6 °C; and when the input flow rate of the cooling water was 0.02 m/s, the GMM rod temperature was 29.5 °C. These figures indicate that at a low cooling water flow rate, the heat generated by the driving coil can be transmitted to the GMM rod. When the cooling water flow rate increases, the heat generated by the driving coil is removed by the cooling water. The temperature of the GMM rod is completely determined by the temperature of the cooling water. In this case, if the temperature continues to increase, the water flow rate of the cooling water flow rate has no effect on the temperature distribution in the GMM rod.

### Experimental investigation

**Figure 12:**
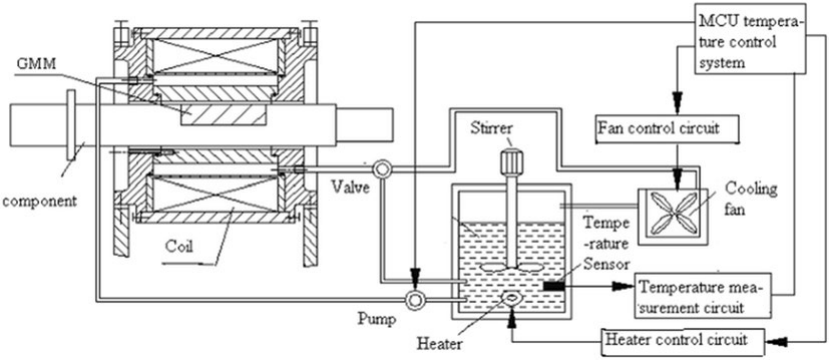
Schematic diagram of new control system for GMM smart component.

The control system of the GMM smart component is designed based on the simulation results. To reduce the effect of the heat generated by the drive coil and improve the accuracy of radial displacement required by the GMM smart components, a new forced water cooling control strategy is proposed, as shown in Fig. 12.

The temperature control system starts heating the water when the temperature is lower than the design threshold. The water pump starts when the water temperature is at the design threshold. While the GMM smart component is working, the temperature of the water tank will rise because the cooling water absorbs heat from the driving coils. When the temperature of the water tank is higher than the design threshold, the temperature control system will switch on the cooling fan. This control strategy is simple because it ensures a constant cooling water temperature. The temperature control system is not required to change the pump velocity according to the temperature.

**Figure 13:**
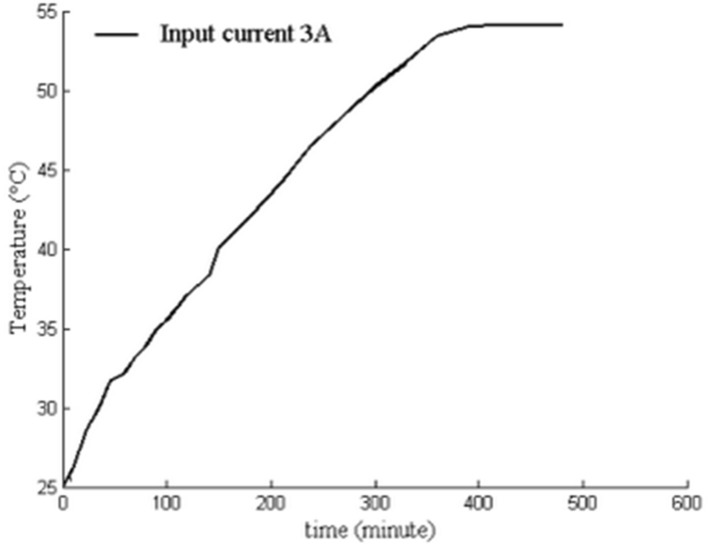
For current 3 A, temperature of GMM smart component rised with time is observed under no cooling water.

The experimental results of the GMM smart component temperature are shown in Fig. 13. The temperature of the GMM smart component increased for 450 min and then reached thermal equilibrium with the temperature remaining constant for a current of 3 A. The temperature of the GMM smart component attained 54.1 °C. The simulation results shown in Fig. 8 and our experimental results show that the model error is less than 0.9%.

**Figure 14:**
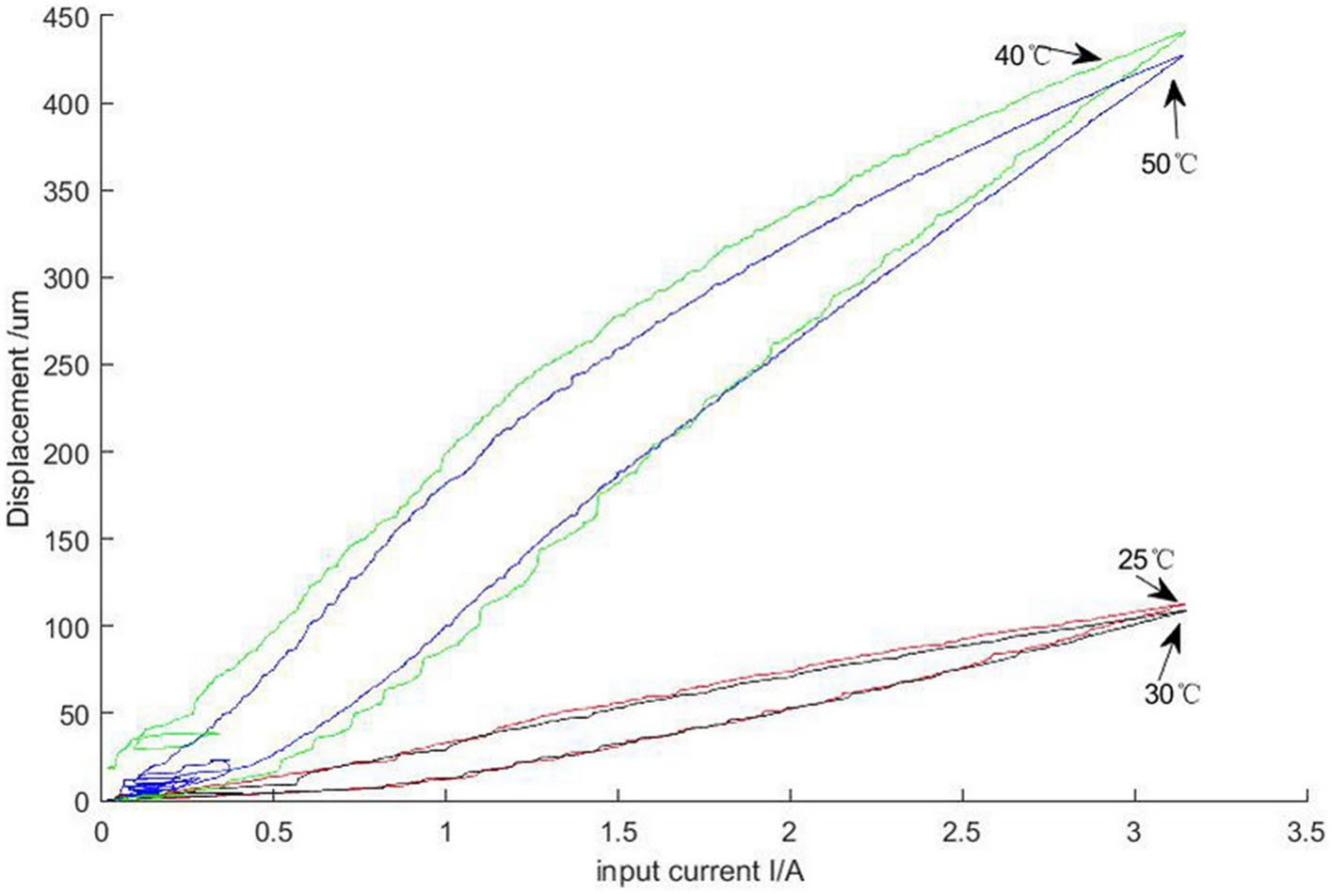
The radial bending deformation of GMM smart component vared with currents under various temperature cooling water.

The experimeantal results of the GMM smart components for different input currents and the radial bending deformation at 25, 30, 40 and 50 °C cooling water are shown in Fig. 14.When the cooling water temperature rises from 25 to 30 °C, the radial bending deformation of GMM smart components with current decreases, but the decrease amount is very small, and the two are basically the same. When the temperature rises from 30 to 40 °C, the radial bending deformation of the GMM smart component increases. when the temperature rises from 40 to 50 °C, the radial bending deformation of the GMM smart component decrease.The maximum radial bending deformation of the component occures at 40 °C cooling water and is 441 μm.

Table 3 shows a comparison of the simulation and experimental results for an ambient temperature of 25 °C and input currents of 0.5, 2, and 3 A using 30 °C cooling water to cool the GMM smart component. The cooling water flow rate was 0.0464 m/s. After 10 min of cooling, the temperature of the GMM smart component was measured.

**Table 3:** Comparison of simulation results and experimental results at different input currents.

Input current (A)	Simulation result (°C)	Measurement result (°C)
0.5	30	30.1
2	30	30.3
3	30	30.2

Table 3 shows that the difference between the simulation result and measured value does not exceed 0.3 °C, and the maximum error is 1%.

Furthermore, Table 4 shows a comparison of the simulation and experimental results for an ambient temperature of 25 °C and cooling water flow rates of 0.01, 0.02, and 0.0464 m/s using 30 °C cooling water to cool the GMM smart component. The input current was 3 A. After 10 min of cooling, the temperature of the GMM smart component was measured.

**Table 4:** Comparison of simulation results and experimental results at different cooling water flow rates.

Cooling water flow rate (m/s)	Simulation results (°C)	Measurement results (°C)
0.01	30	30.4
0.002	30	30.2
0.0464	30	30.1

Table 4 shows that the difference between the simulation result and the measured value does not exceed 0.4 °C, and the maximum error is 1.33%. A comparison of the simulation results and experimental results illustrates that the flow-thermal coupling model of the GMM smart component is correct, but it also shows that the selected temperature control is effective.

## Conclusions

Focusing on the impact of the thermal effect on the performance of GMM smart components, a new simplified control strategy is proposed to ensure a constant GMM temperature. Our study can be summarized as follows:

1. Our strategy involves establishing and implementing a turbulent flow-thermal coupling finite element model for the GMM smart component by applying flow-thermal coupling theory.
2. Our strategy only involves maintaining the cooling water at a certain temperature and does not control the flow rate to eliminate the effects of the drive coil heating elements for precise positioning of the GMM smart component.
3. The control system of the GMM smart component is developed and implemented. When the input current is 3 A, the GMM smart component temperature attains 54.1 °C, while the simulation result is 53.629 °C. The error is within 0.9%. Thus, the experimental results indicate that the flow-thermal coupling model for the GMM smart component is appropriate.
4. When the input current of the GMM smart component is 0.5, 2, or 3 A, the cooling water flow rate is 0.0464 m/s. The cooling water is used to cool the component. The difference between the simulation result and actual measurement does not exceed 0.3 °C; thus, the maximum error is 1%. When the input current is 3 A, the cooling water flow rates are 0.01, 0.02, and 0.0464 m/s, with 30 °C cooling water used to cool the GMM smart component. The difference between the simulation result and the measured value does not exceed 0.4 °C; thus, the maximum error is 1.33%. This indicates that the selected temperature control is effective.

However, the entire temperature-rise control system has a large volume structure, which is not suitable for the application of GMM devices in a narrow space. Therefore, it is necessary to further explore the temperature rise control method using simple structures with a small volume, which would ensure that the GMM device could be more widely used.
